# The left inferior frontal gyrus is involved in adjusting response bias during a perceptual decision-making task

**DOI:** 10.1002/brb3.223

**Published:** 2014-03-15

**Authors:** Greg E Reckless, Olga T Ousdal, Andres Server, Henrik Walter, Ole A Andreassen, Jimmy Jensen

**Affiliations:** 1 Division of Mental Health and Addiction, KG Jebsen Centre for Psychosis Research, Oslo University HospitalOslo, Norway; 2 Department of Clinical Medicine, University of OsloOslo, Norway; 3 Department of Neuroradiology, Oslo University HospitalOslo, Norway; 4 Department of Psychiatry and Psychotherapy, Charité UniversitätsmedizinBerlin, Germany; 5 Centre for Psychology, Kristianstad UniversityKristianstad, Sweden

**Keywords:** fMRI, inferior frontal gyrus, motivation, perceptual decision-making, signal detection theory

## Abstract

**Introduction:**

Changing the way we make decisions from one environment to another allows us to maintain optimal decision-making. One way decision-making may change is how biased one is toward one option or another. Identifying the regions of the brain that underlie the change in bias will allow for a better understanding of flexible decision-making.

**Methods:**

An event-related, perceptual decision-making task where participants had to detect a picture of an animal amongst distractors was used during functional magnetic resonance imaging. Positive and negative financial motivation were used to affect a change in response bias, and changes in decision-making behavior were quantified using signal detection theory.

**Results:**

Response bias became relatively more liberal during both positive and negative motivated trials compared to neutral trials. For both motivational conditions, the larger the liberal shift in bias, the greater the left inferior frontal gyrus (IFG) activity. There was no relationship between individuals' belief that they used a different strategy and their actual change in response bias.

**Conclusions:**

The present findings suggest that the left IFG plays a role in adjusting response bias across different decision environments. This suggests a potential role for the left IFG in flexible decision-making.

## Introduction

Flexibility in the way we make decisions allows us to adapt to changing environments. In one aspect of perceptual decision-making, we make choices about the presence of stimuli in our environment—for example, cues that signal reward or danger. Decision theory suggests that decisions are made through a process whereby sensory evidence is accumulated and compared against a decision criterion (Gold and Shadlen 2007; Deco et al. 2013). The decision criterion is a threshold that determines how much sensory evidence is needed before a stimulus is judged to be present. If accumulated sensory evidence meets the decision criterion, a stimulus is decided to be present, if not, it is judged to be absent. Changes in the decision criterion and the corresponding level of sensory evidence required before a stimulus is judged to be present allow for flexible decision-making (Green and Swets 1966; Bogacz et al. 2006; Ratcliff and McKoon 2008). As behavior, such as approaching a potential reward or avoiding potential danger, follows from the decisions we make, flexible decision-making can lead to flexible behavior. For example, in a decision environment where there is a high probability of reward it would be beneficial to adopt a decision criterion that is biased toward judging reward cues as present. However, if a similarly biased decision criterion was used in an environment where there was a low probability of reward, many reward predicting cues would erroneously be judged to be present and energy would be needlessly expended pursuing rewards that do not exist. Flexible decision-making is, therefore, important for optimizing behavior. Using signal detection theory, the decision criterion can be quantified in terms of *response bias* (how likely an individual will say a stimulus is present), and the change in response bias between decision environments can be measured (Green and Swets 1966; Macmillan and Creelman 2009).

There are several ways that the decision environment may change including how frequently a stimulus is present (base rate) or is expected to be present (prior expectation), the costs and benefits associated with incorrectly and correctly identifying a stimulus (payoff matrix), and the degree to which an individual is motivated to identify a stimulus (motivation). Response bias has been demonstrated to adapt to all four types of changes in the decision environment (Henriques et al. 1994; Maddox and Bohil 1998; Bohil and Maddox 2001; Taylor et al. 2004; Fleming et al. 2010; Forstmann et al. 2010; Summerfield and Koechlin 2010; Reckless et al. 2013). In a rewarded memory task, Taylor and colleagues (Taylor et al. 2004) demonstrated that as the payoff matrix changed, participants altered their response bias to maintain a strategy that optimized the amount of money that could be won. Motivation similarly affects response bias. In a recent perceptual decision-making study, we reported that when motivated, individuals adopted a more liberal response bias, that is, they were more likely to say a target stimulus was present, compared to when they were relatively less motivated (Reckless et al. 2013). This was in keeping with findings from a verbal recognition task, where participants adopted a more liberal response bias when motivated compared to when unmotivated (Henriques et al. 1994).

Both animal electrophysiological and human imaging studies have identified brain regions involved in accumulating and comparing sensory evidence (Binder et al. 2004; Heekeren et al. 2004; Pleger et al. 2006); however, the region or regions which adjust the decision criterion from environment to environment have not been thoroughly investigated. Two possible candidate regions emerge. Heekeren and colleagues (Heekeren et al. 2004, 2006) have suggested that the left superior frontal sulcus (SFS) is involved in comparing accumulated sensory evidence for different choices. In a face-house discrimination task, they found that activation in the left SFS varied with the difference in signal between regions of the brain representing face and house evidence. It was further found that disruption of this region using transcranial magnetic stimulation affected the rate at which sensory evidence was integrated as well as decision accuracy (Philiastides et al. 2011). Given that the left SFS is involved in handling the comparison of sensory evidence, it is possible that this region is also involved in adjusting how much evidence is needed before a decision is made—the role of the decision criterion. Rahnev and colleagues (Rahnev et al. 2011), while examining the effect of prior expectations on visual discrimination, found that the more an individual became biased to a particular choice in response to a predictive cue, the greater the activation in the left inferior frontal gyrus (IFG). Reckless and colleagues (Reckless et al. 2013) similarly found a relationship between a motivation-induced shift toward a more liberal response bias and increased left IFG activation. However, the block design of their study limited the interpretability of this relationship. While both the left SFS and IFG appear to be good candidates for modifying the decision criterion between environments, the relationship between these regions and flexibility in response bias has not been fully explored.

This study aims to identify brain regions involved in adjusting response bias and to determine whether individuals are aware of any change in response bias from one decision environment to another. To this effect, different levels of motivation (positive, negative, and neutral) were used to affect a change in response bias during a perceptual decision-making task where participants were asked to detect a picture of an animal amongst distractors. We proposed two criteria which had to be met for a region of the brain to be considered to play a role in response bias. First, there had to be a relationship between change in activity in the region from one motivational condition to another and the corresponding change in response bias. Second, regardless of whether motivation was positive or negative, the relationship between the change in response bias and activation had to be the same. Further, to examine whether there was a relationship between the participants' belief that they changed bias and their actual change in response bias, they were asked questions concerning the strategies that they used in the different motivational conditions. On the basis of previous findings, we hypothesized that participants would adopt a more liberal response bias (i.e., would be more likely to say the stimulus was present) in the positive and negative motivation conditions compared to the neutral conditions. We further hypothesized that there would be a relationship between change in response bias and activation in the left IFG.

## Materials and Methods

### Participants

Twenty-eight healthy participants were recruited for the study in accordance with local ethics committee guidelines and provided written informed consent. Prior to participation, all subjects were screened and were excluded if they presented with neurological or psychiatric illness, substance abuse, or MR-incompatibility. Subjects were paid NOK 300 ($50) for their participation and kept any additional money they won in the task described below. Four participants were excluded from analyses because they were unable to detect signal from noise (*d*′ ≤ 0). Analysis was performed using data from the remaining 24 subjects (mean age ± SD = 25.3 ± 5.6 years; 15 women; one left-handed).

### fMRI task

A perceptual decision-making task with three different motivational conditions (positive, negative, and neutral) was used to examine the neural correlates of change in response bias. The experiment was composed of two scanning sessions; the positive session (*positive*) included positive (Pos) and neutral trials (Neut-P), and the negative session (*negative*) included negative (Neg) and neutral trials (Neut-N). The inclusion of separate neutral conditions allowed for the examination of any differential effect of positive and negative motivation on their neutral conditions. The task was an event-related, within-subject design where participants performed 34 trials in each of the four conditions. Trials within sessions were presented randomly and the order of sessions was counterbalanced.

**Figure 1:**
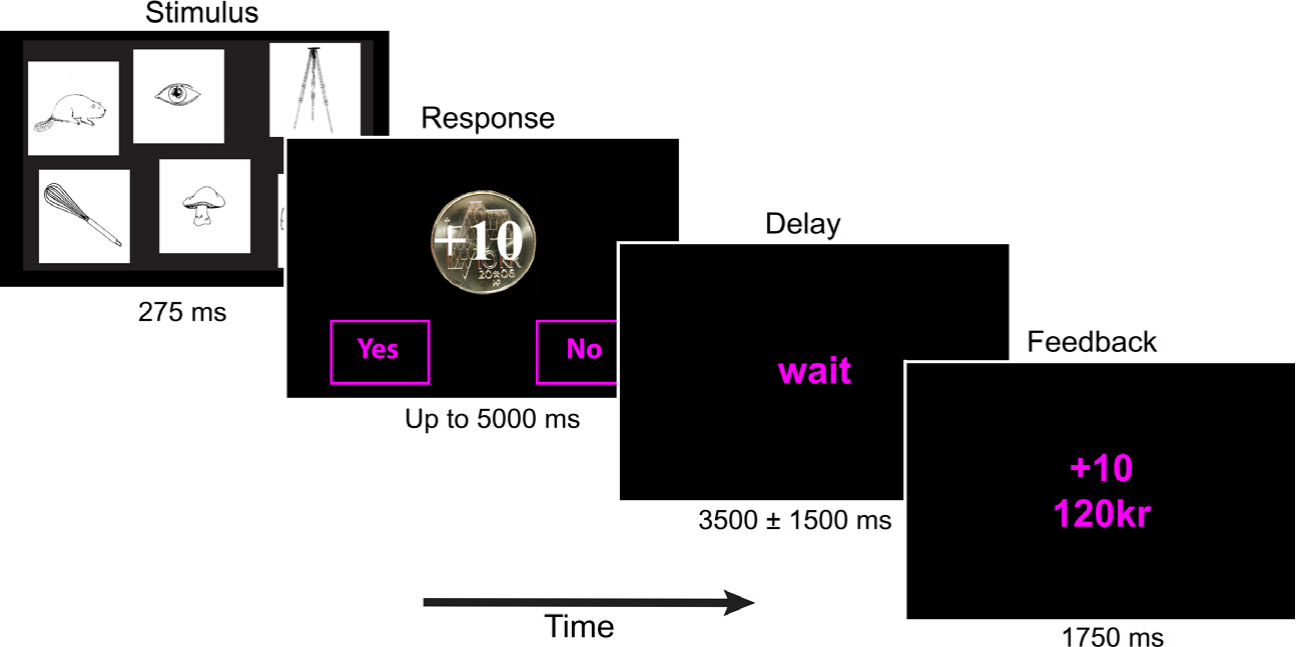
Experimental task. Participants viewed six black and white drawings for 275 msec. A decision screen indicating the amount of money at stake on that trial immediately followed. A gold coin with “+10kr” indicated that 10kr could be won for correct responses and no money would be lost for incorrect responses. On Neg trials the coin was superimposed with “−10kr” and on Neut-P and Neut-N trials the coin was replaced with a white disk. Participants had up to 5 sec to make their response. A delay screen was presented for a jittered duration of 3.5 ± 1.5 sec immediately following a decision. Upon termination a feedback screen depicting the money obtained on that trial and the total amount won up to that point was presented (1750 msec). Trials were separated with a jittered ITI of 5 ± 2 sec.

Each trial began with a screen depicting six black and white line drawings (275 msec) (Snodgrass and Vanderwart 1980)^1999^ (Fig. 1). Participants then viewed a screen cueing motivational condition and had up to 5 sec to indicate with a button press using the index finger of one hand whether one of the six pictures depicted an animal. The index finger of the other hand was used to indicate if an animal was not present. Handedness was counterbalanced across participants. The motivational cue appeared after the stimulus to isolate the effect of motivation on decision behavior and to avoid the confounding effect of motivation mediated increases in perceptual processing through mechanisms such as attention (Engelmann and Pessoa 2007; Engelmann et al. 2009; Pessoa 2009). Positive motivation trials were cued by a gold coin with “+10kr” superimposed. Here, 10kr ($1.50) could be won for correct responses (hits and correct negatives) and no money would be lost for incorrect responses (misses and false positives). Negative trials were cued with the same gold coin with an orange tint and “−10kr” superimposed. On these trials, no money would be won for correct responses, but 10kr would be lost for incorrect responses. The tinting of the coin was counterbalanced across participants. Neutral trials where no money could be won or lost were cued by a white disk the same dimensions as the coin. A jittered delay (3.5 ± 1.5 sec) separated the participants' decision from a feedback screen (1750 msec) which depicted the amount of money obtained on that particular trial as well as the total amount of money that had been gained so far. As no money could be won or lost on neutral trials, only the total amount of money was displayed on the feedback screen. Individual trials were separated by a jittered intertrial interval lasting 5 ± 2 sec.

Participants completed a practice version of the task outside of the scanner to limit learning effects. The practice task was identical to the experimental task except that the target stimuli were modes of transportation instead of animals. The images used in the practice task were not included in the experimental task.

### Apparatus

The paradigm was programmed and controlled using E-Prime software (version 1.2; Psychology Software Tools, Inc.; Pittsburgh, PA, USA). Stimuli were presented to the participants in the scanner using VisualSystem (NordicNeuroLab, Bergen, Norway) and responses were collected using ResponseGrips (NordicNeuroLab).

### Image acquisition

Whole-brain, T2*-weighted, echo-planar images (TR = 2 sec; TE = 25 msec; FA = 90°) were acquired using a GE Signa HDx 3T scanner with a standard eight-channel head coil (General Electric Company; Milwaukee, WI, USA). Each volume consisted of 36 slices acquired parallel to the AC-PC plane (sequential acquisition; 3.5 mm thick with a 0.5 mm gap; 260 × 260 mm in-plane resolution, 64 × 64 matrix). The first three volumes were discarded to allow for magnetization equilibrium. A T1-weighted FSPGR structural image (TR = 7.7 msec, TE = 3.0 msec, flip angle 12°) was acquired for anatomical comparison. Cushions were placed around the participants' head to minimize movement and earplugs and headphones were used to minimize noise.

### Behavioral analysis

#### Effect of motivation on decision-making behavior

Discrimination *(d′)* and response bias (*c*) were calculated using signal detection theory (Macmillan and Creelman 2009). Discrimination measures one's ability to identify a target stimulus from a nontarget stimulus and is calculated using the inverse *z*-transformed hit rate (HR) and false-positive rate (FPR):


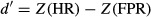


A *d′* score of 0 indicates an inability to discriminate between stimuli. The better an individual's discrimination, the larger the *d′* score. Response bias is calculated as:


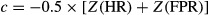


and measures a participant's willingness to say the target stimulus is present. A response bias equal to 0 indicates that a participant is equally likely to say a target or nontarget stimulus is present. A larger positive score indicates that the participant is less likely to say the target stimulus is present (*conservative bias*), while a large negative score indicates an increased willingness to say the target stimulus is present (*liberal bias*). Given the equal proportion of target and nontarget trials and the neutral payoff matrix in this study, the mathematically optimal response bias is neutral (*c = 0*).

Two one-way, repeated-measures ANOVAs were used to test the effect of motivation on discrimination and response bias (IBM SPSS Statistics for Windows, Version 19.0. Armonk, NY, USA: IBM Corp.). A two-way (4 × 2), repeated-measures ANOVA was used to examine the effect of motivation and decision (Yes/No) on the natural log (ln) transformed response times (RT). Greenhouse–Geisser corrections were applied when the assumption of nonsphericity was broken. Significant differences were identified at *P* < 0.05. Effect sizes were calculated using Pearson's *r*. Values of *r* = 0.10, 0.30, and 0.50 reflect small, medium, and large effect sizes, respectively (Cohen 1988).

Where there was a significant difference in response bias between levels of motivation, the change in response bias (Δ*c*) was calculated as:


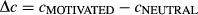


The more negative Δ*c*, the bigger the shift toward a more liberal response bias. The more positive Δ*c*, the bigger the shift toward a more conservative response bias.

To examine how participants' belief about their change in strategy related to their actual change in response bias, immediately after completing the paradigm and while still in the scanner, they used the response grips to indicate on a 10-point scale ranging from one to 10 whether they “used a different strategy when they could win 10kr compared to when they couldn't win or lose any money” and whether they “used a different strategy when they could lose 10kr compared to when they couldn't win or lose any money.” The scale was anchored at each end with the qualifiers “not at all” and “very much so.” Spearman's correlations (*r*_*s*_) were performed between the question scores and the absolute value of the change in response bias (|Δ*c*|). The absolute value of the change in response bias was used because it gives a measure of the magnitude of the change in response bias regardless of the direction of the change.

### fMRI analysis

Data preprocessing and image analysis were conducted using Statistical Parametric Mapping (SPM8, http://www.fil.ion.ucl.ac.uk/spm/; Wellcome Trust Centre for Neuroimaging, London, UK). Motion was assessed using the TSDiffANA toolbox (http://sourceforge.net/projects/spmtools/), and no participants were found to have moved more than 3 mm in any direction. All volumes were realigned to the first volume (Friston et al. 1994), and the mean functional and anatomical images were coregistered. The images were then spatially normalized to the Montreal Neurological Institute (MNI) EPI template (Evans et al. 1992), resampled to a voxel size of 3 × 3 × 3 mm, and smoothed using a 8 mm full-width at half-maximum Gaussian kernel. A high-pass filter using a cut-off value of 128 sec and the SPM8 AR1 function were applied.

The data were analyzed by modeling three event types (stimulus, decision, and feedback) as stick functions convolved with a synthetic hemodynamic response function. The three events were specified for “yes” and for “no” decisions for each motivational condition. The six motion parameters estimated during realignment were entered into the model as multiple regressors. The stimulus and decision events were combined and contrasted against an implicit baseline at the first level. These contrast images were moved up to a second level, random-effects, flexible–factorial model where the effects of negative (Neg > Neut-N) and positive (Pos > Neut-P) motivation as well as any differences between neutral conditions (Neut-N > Neut-P; Neut-P > Neut-N) were examined. Significant clusters were identified at *p*_*FWE*_ < 0.05 (family-wise error corrected), *k* ≥ 10 (extent threshold). Activations were localized to a particular anatomical region using the SPM anatomy toolbox (Eickhoff et al. 2006, 2007).

To identify regions where activity correlated with change in response bias, a second–level, linear regression model specifying the positive motivation contrast images (Pos > Neut-P) and the change in response bias (Δ*c*_positive_) as a covariate was used. A whole-brain analysis identified significant clusters at *p*_*FWE*_ < 0.05, *k* ≥ 10. As we had a priori interest in the left SFG and IFG, a region-of-interest (ROI) analysis was also performed. A mask of these regions created by Nielsen and Hansen (Nielsen and Hansen 2004) using probability density estimates from the BrainMap database (Fox and Lancaster 1994) was applied to the contrast image. Small volume correction using a threshold of *p*_*FWE*_ < 0.05, *k* ≥ 10 was then used to identify significant clusters within the masked region. A linear regression was also performed for the negative motivation contrast (Neg > Neut-N) and (Δ*c*_negative_) as a covariate.

## Results

### Behavioral

**Figure 2:**
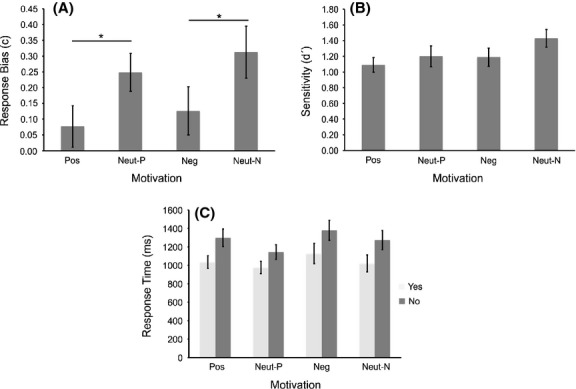
Effect of motivation on perceptual decision-making behavior. Both positive and negative motivation significantly affected response bias (A) with participants more likely to respond that the target stimulus was present in the motivated condition compared to the neutral condition. There was no effect of motivation on either detection sensitivity (B) or response time (C). *P < 0.05.

Motivation did not significantly affect participants' ability to discriminate between target and nontarget stimuli [*F*_(3,69)_ = 2.48, *P* = 0.07] (Table 1, Fig. 2A). It did affect response bias [*F*_(3,69)_ = 4.13, *P* = 0.01]. Pairwise comparisons revealed that participants adopted a more liberal response bias in the positive and in the negative motivation conditions compared to their respective neutral conditions (mean ± SD) [0.08 ± 0.32 vs. 0.25 ± 0.29, *P* = 0.03, *r* = 0.44] and [0.13 ± 0.37 vs. 0.31 ± 0.41, *P* = 0.03, *r* = 0.45] respectively (Table 1, Fig. 2B). On a 10-point scale anchored by “not at all” to “very much so” participants rated their change in strategy as 3.5 (4.8) (median [interquartile range]) in the positive session and 3.5 (6.5) in the negative session. There was no significant correlation between the strength of participants' belief that they used a different strategy and the magnitude of their change in response bias for either positive (*r*_*s*_ = 0.24, *P* = 0.25) or negative motivation (*r*_*s*_ = −0.17, *P* = 0.44).

**Table 1:** Behavioral measures

	Pos	Neut-P	Neg	Neut-N
Response bias (c)	0.08 ± 0.32	0.25 ± 0.29	0.13 ± 0.37	0.31 ± 0.40
*d*-prime (*d*′)	1.09 ± 0.45	1.20 ± 0.65	1.19 ± 0.57	1.43 ± 0.55
Response time (msec)	1171 ± 389	1064 ± 339	1260 ± 502	1158 ± 448

Values are reported as mean ± standard deviation.

Motivation did not have a significant effect on response time [*F*_(1.21,27.74)_ = 3.41, *P* = 0.07], however, decision did [*F*_(1, 23)_ = 50.92, *P* < 0.001, *r* = 0.83] (Table 1, Fig. 2C). “Yes” decisions were significantly faster than “no” decisions (974 msec [95% CI 855–1109 msec] vs. 1194 msec [95% CI 1035–1377 msec]) (Fig. 2D). There was no interaction between motivation and decision [*F*_(3,69)_ = 0.74, *P* = 0.53].

As there is a known trade-off between speed and accuracy in forced choice, perceptual decision-making (Bogacz et al. 2006, 2010), a post hoc analysis was performed to investigate the effect difference in response time (RT) for “yes” and “no” responses had on accuracy. A paired sample *t*-test revealed that “yes” decisions resulted in more correct response than “no” decisions [*t*_(23)_ = 3.30, *P* = 0.003, *r* = 0.57]; (75.8 ± 8.0% [mean ± SD] vs. 70.4 ± 7.7%), respectively.

### Imaging

#### Effect of positive and negative motivation

Whole-brain analyses found that positive motivation (Pos > Neut-P) resulted in significantly greater activation in the bilateral ventral striatum (VS), right IFG, bilateral middle occipital gyrus (MOG) compared to its neutral condition (Table 2). Negative motivation (Neg > Neut-N) resulted in greater bilateral VS, left ventral tegmental area, right fusiform gyrus, and left MOG activation when contrasted with its corresponding neutral condition (Table 2). There were no significant differences between the neutral conditions (Neut-N > Neut-P and Neut-P > Neut-N).

**Table 2:** Effect of motivation on BOLD activity: fMRI whole-brain analysis

Region	Laterality	*x*	*y*	*z*	Peak *z*-score	*p*_FWE_
Pos > Neut-P						
Ventral striatum	Right	9	14	−8	5.93	<0.001
	Left	−9	14	−8	4.71	<0.05
Inferior frontal gyrus	Right	30	26	−14	4.70	<0.05
Middle occipital gyrus	Right	30	−91	7	7.31	<0.001
	Left	−18	−100	4	6.83	<0.001
Neg > Neut-N						
Ventral striatum	Left	−9	14	−8	5.29	<0.005
	Right	9	14	−5	4.96	<0.01
Ventral tegmental area	Left	−3	−25	−11	4.73	<0.05
Fusiform gyrus	Right	24	−85	−8	6.90	<0.001
Middle occipital gyrus	Left	−21	−97	7	6.55	<0.001

Family-wise error correction *p*_*FWE*_ < 0.05, *k* = 10. Only clusters with >10 voxels reported. Anatomical region, hemisphere and coordinates are based on the Montreal Neurological Institute (MNI) labeling system.

#### Correlation between change in response bias and brain activation

**Figure 3:**
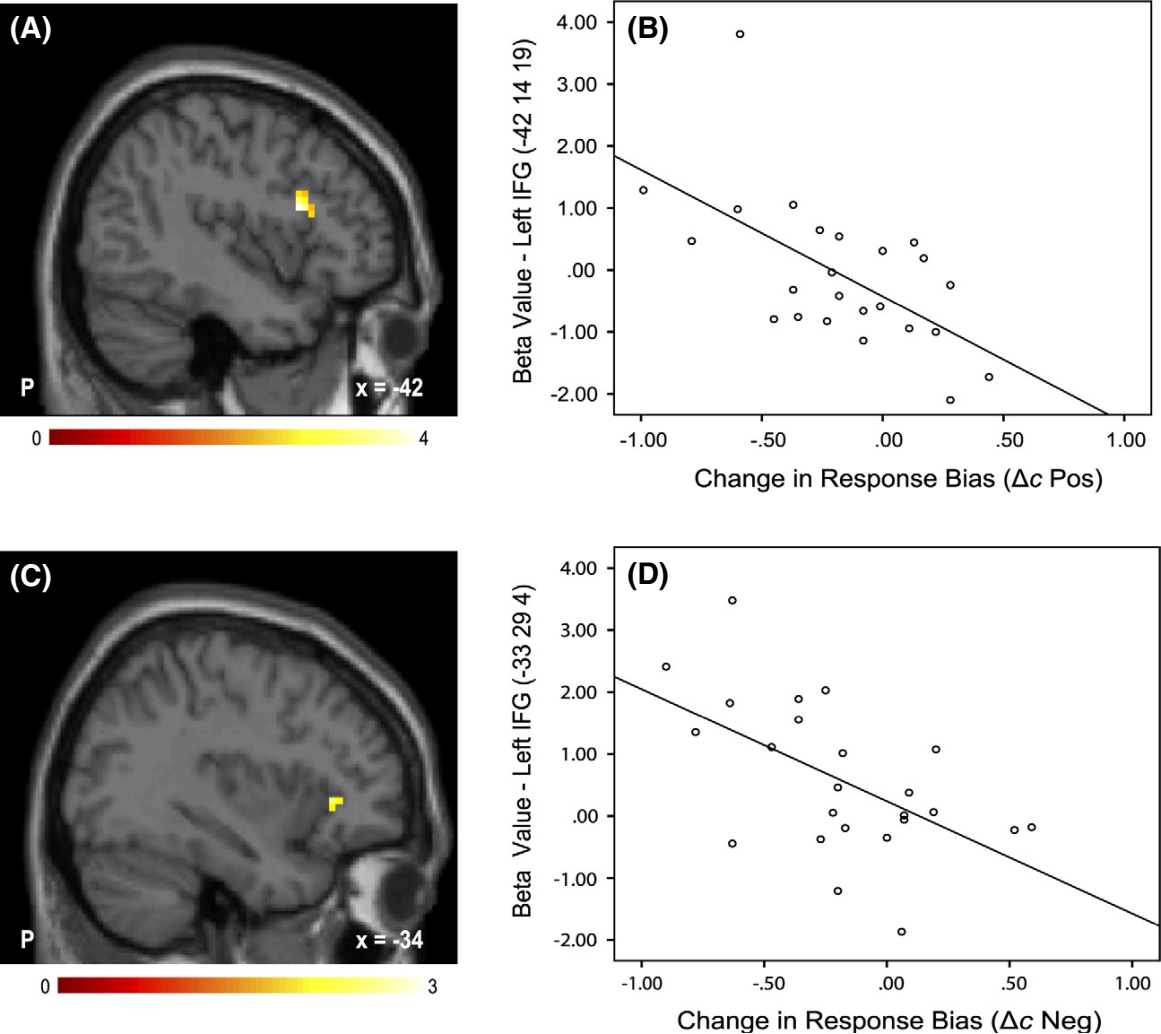
Correlation between the change in response bias and activation in the left IFG. The larger the shift toward a liberal response bias (Δ*c*), the greater the left IFG activation for both the Pos compared to Neut-P (A & B) and Neg compared to Neut-N (C & D) conditions.

Region-of-interest analyses revealed that the shift to a more liberal response bias in the positive motivation condition (Δ*c*_Positive_) correlated with increased activation in the left IFG *pars triangularis* (MNI coordinates: x, y, z: −42, 14, 19; *r* = −0.67, *p*_*FWE*_ < 0.05) (Pos > Neut-P) (Fig. 3A and B). Similarly, in the negative motivation condition, increased activation in the left IFG *pars triangularis* (MNI coordinates: *x*, *y*, *z*: −33, 29, 4; *r* = −0.62, *p*_*FWE*_ < 0.05) (Neg > Neut-N) correlated with the liberal shift in response bias (Δ*c*_Negative_) (Fig. 3C and D). Whole-brain analyses did not identify any additional regions.

## Discussion

Using response bias as a measure for decision criterion and altering it by manipulating motivation in a perceptual decision-making task, the left IFG was identified as a possible response bias regulating region. This region met the two criteria we established a priori: BOLD activity correlated with the change in bias from the neutral to the motivated conditions, and this relationship held true regardless of whether positive or negative motivation induced the shift in response bias.

In line with previous findings (Henriques et al. 1994; Reckless et al. 2013), motivation resulted in the adoption of a more liberal response bias compared to when less motivated. There was, however, no motivation mediated increase in detection sensitivity. While the absence of such a relationship is in keeping with results from a study using a similar paradigm (Reckless et al. 2013), it is contrary to other perceptual decision-making studies that suggest a positive, linear relationship between motivation and increased performance (Engelmann and Pessoa 2007; Engelmann et al. 2009). These studies, however, used a discrimination task while this study used a detection task. Still, the absence of a relationship between motivation and performance draws into question whether the flexibility in decision-making observed in this study was actually adaptive. Response bias, however, was mathematically more optimal in the motivated conditions. This means that when individuals had the opportunity to win money or avoid losing money they adopted a response bias that would most likely allow them to accomplish this goal. This suggests that the change in response bias was indeed adaptive.

The present results showed that “yes” decisions were significantly faster than “no” decisions. Given that there is a known trade-off between speed and accuracy in forced-choice, perceptual decisions (Binder et al. 1999; Huettel et al. 2004; Wenzlaff et al. 2011), and that participants were biased toward “yes” choices in the motivated conditions, it was important to establish whether there was a general change in decision-making strategy between motivational conditions beyond the motivation-mediated change in bias. Although faster, “yes” decisions resulted in significantly more correct responses than “no” decisions. This is contrary to the established trade-off between speed and accuracy where slower decisions are more accurate than fast decisions (Binder et al. 1999; Huettel et al. 2004; Wenzlaff et al. 2011). The absence of an interaction between decision type and motivation indicates that “yes” responses were faster than “no” responses in all conditions. This then excludes a possible confound of a more general strategy shift on change in response bias and its corresponding changes in brain activity. It is possible that the faster, “yes” responses reflect immediate identification of the animal target, while the slower “no” responses are driven by the continuing search for a target that is not present. The combined behavioral results suggest that motivation induced a change in response bias that was adaptive and that the change in bias was not confounded by another more general change in strategy.

The IFG met the two criteria proposed a priori—its activity correlated with the change in bias between motivational conditions, and the relationship held true regardless of the valence of motivation that drove the shift in response bias This region has previously been implicated in the choice between alternatives (Zhang et al. 2004; Moss et al. 2005). For example, Zhang and colleagues (Zhang et al. 2004) found increased activation in the left IFG when participants viewed a cue that indicated that they must choose between two sets of letters compared to when they viewed a cue indicating they did not have to make a choice. It has also been suggested that the left IFG is involved in switching between rules that guide choice selection (Crone et al. 2006; Philipp et al. 2013). During a task where participants were cued as to which choice rule to use when observing a subsequent target, Crone and colleagues (Crone et al. 2006) found that there was greater left IFG activation during trials that required participants to switch to a different choice rule. This study's finding that left IFG activation correlated with the change in response bias for both positive and negative motivation is in accordance with the region's previously observed role in choice selection and rule switching. Response bias measures the decision criterion (Green and Swets 1966; Macmillan and Creelman 2009). That is, it is a quantification of the rule that determines how a choice is made. When response bias shifted to a relatively more liberal bias, that is, the decision criterion changed, increased activation was observed in the left IFG. This seems to be in line with the aforementioned findings from Crone and colleagues (Crone et al. 2006) who observed an increase in activity in this region when the rule used to make a choice needed to be changed.

Previous studies investigating the neural correlates of perceptual decision-making have implicated the left SFS in the computation of perceptual decisions (Heekeren et al. 2004, 2006; Pleger et al. 2006; Philiastides et al. 2011). For example, Heekeren and colleagues (Heekeren et al. 2006) found that activation in the left superior frontal sulcus reflected the comparison of accumulated evidence needed for the discrimination of perceptual stimuli. When activity in this region was disrupted, the rate of sensory evidence accumulation decreased and decisions became less accurate (Philiastides et al. 2011). While the left SFS may be involved in comparing sensory evidence, we (Reckless et al. 2013) previously found that a more ventral region of the frontal cortex, the left IFG may be involved in adjusting the decision criterion between different decision environments. However, the block design of that study limited how this relationship could be interpreted. The present findings suggest that the left IFG is indeed involved in adapting the decision criterion. This is in keeping with findings from Rahnev and colleagues (Rahnev et al. 2011) who found that individuals who adjusted their response bias more based on information from predictive cues had greater activation in the left IFG. However, one problem that arises when considering results across perceptual decision-making studies is whether the perceptual decision-making task was one of detection or one of discrimination and what decision-making model was used to evaluate the behavioral and imaging findings. This study used a detection task and signal detection theory (SDT). Rahnev and colleagues (Rahnev et al. 2011), who found a similar relationship between response bias and activation in the left IFG, used a discrimination task and SDT. The finding that there is a relationship between response bias and the left IFG in both a discrimination and a detection task suggests that the relationship is independent of the type of perceptual decision-making task performed.

A limitation of this study was that it used one theory of decision-making to investigate the neural correlates of the decision criterion. While the change in response bias was correlated with the change in activation from the positive and negative motivation conditions to their respective neutral conditions, it is unknown whether this pattern is unique to response bias or whether it can be observed using other models of perceptual decision-making. The drift diffusion model (DDM) (Ratcliff 1978; Ratcliff and Smith 2004) of perceptual decision-making has gained in popularity because of its ability to explain observed trade-offs between speed and accuracy. Unlike SDT that suggests a single decision-criterion, DDM suggest two criteria—one for each alternative. These criteria are represented in terms of decision boundaries which, when bias is neutral, lie an equal distance but on opposite sides of a point at which evidence accumulation begins. Here, response bias is modeled as a shift in the starting point toward one decision boundary and away from the other. Perceptual decision-making studies that have used DDM have found that “drift rate,” how fast accumulated evidence approaches one of the decision boundaries, is what seems to be driving the activation in the left SFS (Heekeren et al. 2006; Summerfield et al. 2006; Philiastides et al. 2011). However, Mulder and colleagues (Mulder et al. 2012), using the DDM, found that when they separately manipulated prior probability and payoff matrix in a random dot-motion task, change in bias was associated with increased left IFG activation. In effect, bias toward one decision boundary or another was associated with left IFG activation. This suggests that the relationship between the change in bias and the left IFG activation is not unique to the SDT model of decision-making. The finding that there is an association between a change in the decision criterion in both detection and discrimination studies and that this relationship transcends the model used to investigate it provides converging evidence that the left IFG is involved in adjusting decision criterion between different environments.

## Conclusions

Flexibility in the way we make decisions allows us to maintain an optimal choice strategy as the decision environment changes. Findings from this study suggest that the left IFG contributes to this flexibility through its involvement in adjusting how we bias our choices. Given that subsequent behavior often follows from present decisions, the left IFG may, to some extent, play a role in flexible behavior.
